# Generalized synchronous output of neural populations - does it only encode fast stimulus components?

**DOI:** 10.1186/1471-2202-14-S1-P244

**Published:** 2013-07-08

**Authors:** Alexandra Kruscha, Benjamin Lindner

**Affiliations:** 1 Bernstein Center for Computational Neuroscience, Berlin, 10115, Germany; 2 Institute for Physics, Humboldt-Universität zu Berlin, Berlin, 12489, Germany

## 

Neurons are subject to intrinsic noise, which influences the information transmission of an incoming signal. Which information can we derive from a population of noisy neurons, that are uncoupled, but driven by a common broadband stimulus? Intuitively, one expects that the times where the action potentials of the different neurons coincide (synchronous spikes), contain different information about the common stimulus than the output of a single neuron does.

Indeed, experiments in a electro-sensory model system [1] revealed that synchronous spikes encode preferentially fast (high-frequency) components of the stimulus, i.e. synchrony acts as an information filter. A recent theoretical study [2] confirmed this finding and uncovered the stochastic mechanism of this filter.

To simplify the analytical approach, Ref. [2] used a rather strict measure of synchrony: all neurons in the population have to fire within a short time window. Here we generalize this to a measure of the synchronous output, for which only m out of n neurons in the population have to fire in synchrony. We inspect, how well this measure works for different ratios m/n and present an analytical approach to the spectral coherence function that characterizes the information transfer of the stimulus to the synchronous output. We discuss under which conditions this generalized synchrony acts as a band-pass filter.
